# Genome Mining and Analysis of PKS Genes in *Eurotium cristatum* E1 Isolated from Fuzhuan Brick Tea

**DOI:** 10.3390/jof8020193

**Published:** 2022-02-16

**Authors:** Xiaoxiao Guo, Fusheng Chen, Jiao Liu, Yanchun Shao, Xiaohong Wang, Youxiang Zhou

**Affiliations:** 1 College of Food Science and Technology, Huazhong Agricultural University, Wuhan 430070, China; guoxiaoxiao0321@aliyun.com (X.G.); chenfs@mail.hzau.edu.cn (F.C.); yanchunshao@mail.hzau.edu.cn (Y.S.); 2 Key Laboratory of Environment Correlative Dietology, Ministry of Education, Huazhong Agricultural University, Wuhan 430070, China; 3 Institute of Agricultural Quality Standards and Testing Technology Research, Hubei Academy of Agricultural Sciences, Wuhan 430064, China; babojiao@126.com; 4 Hubei Key Laboratory of Nutritional Quality and Safety of Agro Products, Wuhan 430064, China

**Keywords:** *Eurotium cristatum*, Fuzhuan brick tea, whole genome, polyketide synthase, secondary metabolites, emodin, flavoglaucin

## Abstract

*Eurotium cristatum* as the dominant fungi species of Fuzhuan brick tea in China, can produce multitudinous secondary metabolites (SMs) with various bioactivities. Polyketides are a very important class of SMs found in *E*. *cristatum* and have gained extensive attention in recent years due to their remarkable diversity of structures and multiple functions. Therefore, it is necessary to explore the polyketides produced by *E*. *cristatum* at the genomic level to enhance its application value. In this paper, 12 polyketide synthase (PKS) genes were found in the whole genome of *E*. *cristatum* E1 isolated from Fuzhuan brick tea. In addition, the qRT-PCR results further demonstrated that these genes were expressed. Moreover, metabolic analysis demonstrated *E*. *cristatum* E1 can produce a variety of polyketides, including citreorosein, emodin, physcion, isoaspergin, dihydroauroglaucin, iso-dihydroauroglaucin, aspergin, flavoglaucin and auroglaucin. Furthermore, based on genomic analysis, the putative secondary metabolites clusters for emodin and flavoglaucin were proposed. The results reported here will lay a good basis for systematically mining SMs resources of *E*. *cristatum* and broadening its application fields.

## 1. Introduction

Fuzhuan brick tea is one of traditional microbial fermented teas in China [1]. Historically, it has been a daily necessity for ethnic minorities in China’s Qinghai, Xinjiang, Tibet, Inner Mongolia and other border regions [2]. During the manufacturing process of Fuzhuan brick tea, fungal fermentation is the unique step in achieving particular characteristics and pharmacological health benefits [3]. *E*. *cristatum*, as the dominant fungus involved in this process, can form yellow cleistothecium, which looks like a golden flower, and is therefore commonly known as “Jinhua” by providers and consumers in China [4,5]. Due to the distinctive influence on the color, aroma, and flavor of Fuzhuang brick tea by “Jinhua”, the quality and the amount of “Jinhua” were considered as the important parameters to evaluate the quality of Fuzhuan brick tea [6].

In the 1940s, the first study regarding the identification of the dominant fungi in Fuzhuan brick tea was reported. In 1990, according to the international rules of nomenclature for algae, fungi, and plants, this fungus was identified as *Eurotium cristatum* (anamorph: *Aspergillus spiculosus*, synonym: *Aspergillus* *cristatus*) by observing its morphology under a microscope and scanning electron microscope [7].

The fermentation of *E*. *cristatum* gives Fuzhuan brick tea specific color, aroma, taste, and some useful physiological functions for human health such as anti-hyperlipidemia, anti-obesity, treating cardiovascular diseases, helping digestion, etc. [8,9]. Previous studies implied that these interesting characteristics were ascribed to the secreted SMs from *E*. *cristatum*. However, the mechanisms for these potential functional SMs at the genomic, transcriptional, and metabolic level are not yet clear. 

**Figure 1:**
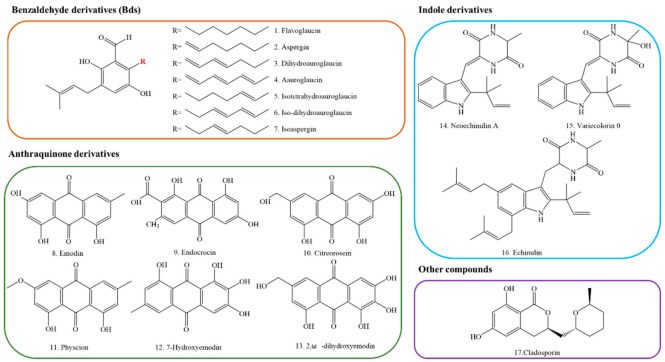
Chemical formulations of some common secondary metabolites in *Eurotium cristatum*.

Fungal polyketides (PKs) are very important secondary metabolites with diverse structures, functional diversities, and a wide range of biological activities [10]. *E*. *cristatum* is a kind of common indoor filamentous fungi, which is renowned for producing multitudinous secondary metabolites with various bioactivities [11]. At least 25 secondary metabolites in *E*. *cristatum* were identified in previous reports [12,13,14,15]. According to their chemical structures, these secondary metabolites can be classified into four types, benzaldehyde derivatives (Bds), anthraquinone derivatives, indole derivatives, and other compounds, respectively (as shown in Figure 1). About 70% of these SMs are polyketides, mainly including benzaldehyde and anthraquinone derivatives. To date, studies on these compounds have only focused on their structures and bioactivities. Therefore, the research on SMs especially PKs produced by *E*. *cristatum* is still in its infancy, and the mining is far from enough. In addition, the genetic and molecular basis for the biosynthesis of these PKs in *E*. *cristatum* is still unclear. 

In this study, the genome of *E*. *cristatum* E1 isolated from Fuzhuan brick tea was obtained, all the PKS genes in this fungus were predicted and the expression level of these genes was analyzed. Then, SMs were detected and identified by UPLC and UPLC-MS/MS. The putative emodin and flavoglaucin biosynthetic gene cluster were proposed, and the comparison with known gene clusters was performed. In general, this study will have important theoretical guiding value for elucidating the diversity of secondary metabolites produced by *E*. *cristatum*, and as far as possible to provide a basis for the development and application of *E*. *cristatum*.

## 2. Materials and Methods

### 2.1. Strains, Growth Conditions and Genomic DNA Extraction

*E*. *cristatum* E1 (CCTCC M20211112) was isolated from Fuzhuan brick tea in Yi Yang City, Hunan Province, China. The fungus was cultured on MYA medium (malt extract powder 20 g, yeast extract powder 5 g, sucrose 30 g, agar 20 g, and water 1000 mL) with cellophane at 28 °C for 3 days. Mycelia were gathered from cellophane and crushed in a mortar with liquid nitrogen. The CTAB method was used to extract the genomic DNA [16].

### 2.2. Genome Sequencing, Assembly and Annotation

The extracted DNA was detected by agarose gel electrophoresis and quantified by FastQC [17]. The genome was sequenced by single-molecule real-time (SMRT) technology. Sequencing was performed at the Beijing Novogene Bioinformatics Technology Co., Ltd. The low-quality reads were filtered by the SMRT Link v5.0.1 [18] and the filtered reads were assembled to generate one contig without gaps.

The Augustus 2.7 program was used to predict the coding gene [19]. Gene function was predicted by using the KOG (Clusters of Eukaryotic Orthologous Groups) database. The genome-wide circle was drawn by using Circos V0.64 software.

### 2.3. Phylogenetic Relationships and Comparative Genomic Analysis

Taxonomy was performed through the construction of a phylogenetic tree based on the alignment of partial β-tubulin (*BenA*), calmodulin (*CaM*) and RNA polymerase II second largest subunit (*RPB2*) genes. Homologous sequences were identified using BlastN against the nr NCBI database [20]. The accession number of used genes is shown in Supplementary Table S1. Sequences were aligned by ClustalW in MEGA 5.05 [21]. All positions containing gaps and missing data were eliminated from the dataset (complete deletion option). Phylogenetic inference was performed in MEGA 5.05 with the Neighbor-Joining approach with 100 bootstrap replicates [21].

Orthologous genes were searched between *E*. *cristatum* E1 and *A*. *cristatus* GZAAS 20.1005 genomes using the OrthoFinder program (version 2.5.4) [22]. The two genomes were aligned with the Progressive Mauve algorithm by using MAUVE (version 2.3.0) [23].

### 2.4. Identification of Biosynthetic Gene Clusters

Biosynthetic gene clusters (BGCs) were analyzed by the antiSMASH 5, using the standard parameters with options of fungal BGC scanning setups [24]. The Known Cluster Blast module was enabled to compare predicted BGCs with those validated and characterized BGCs deposited on the Minimal Information about a Biosynthetic Gene cluster (MIBiG) database [25].

### 2.5. Identification of Polyketide Synthase Genes

The minimal structure of fungal PKS only requires the highly conserved sequences of β-ketosynthase (KS), acyltransferase (AT) and acyl carrier protein (ACP) domains. Thus, the three domains are used in screening for PKSs. Then, the Pfam database [26] and NCBI CDD database [20] were used to scan for significant domains of these proteins. The conserved functional domains present in PKSs were further confirmed using Synthaser (v1.1.0) [27], and the function of these genes was predicted using NCBI BLASTP with an E-value of <1 × 10^−10^ after setting up the online BLAST database [20].

### 2.6. The Relative Expression Level Analysis of PKS Genes

RNA was isolated from mycelia grown on cellophane membranes covering PDA plates using TransGen Biotech TransZol Up Plus RNA Kit (TransGen, Beijing, China). RNA is retrotranscribed into cDNA using Vazyme HiScript II Q RT SuperMix for qPCR (+gDNA wiper) (Vazyme, Nanjing, Jiangsu, China).

Quantitative real-time RT-PCR (qRT-PCR) was performed by qTOWER 2.2 Real-Time PCR Systems (Analytik Jena, Jena, Germany). Relative expression was evaluated with 12.5 μL Vazyme AceQ Universal SYBR qPCR Master Mix (Vazyme, Nanjing, Jiangsu, China), 1.0 μL of 2.5 μM forward primer, 1.0 μL of 2.5 μM reverse primer, and 2.0 μL of template cDNA. Thermal cycling conditions comprised initial denaturation at 94 °C for 1 min followed by 40 amplification cycles at 95 °C for 15 s, 58 °C for 20 s, and 72 °C for 20 s with a final extension step at 72 °C for 5 min [16]. *β-actin* was used as the reference gene. The primers used in this section are listed in Supplementary Table S2.

### 2.7. Analysis of Secondary Metabolites Produced by Eurotium cristatum E1

Two hundred microliters of fresh spore suspensions (10^5^/mL) of *E*. *cristatum* E1 was inoculated on cellophane membranes covering PDA plates and cultivated at 28 °C for 11 days. The mycelia were collected and freeze-dried, and the media were dried to a constant weight in a 37 °C oven. Twenty milligrams of mycelia or 50 mg media was extracted by 1.5 mL 80% methanol with ultrasound for 20 min. After that, the samples were centrifuged (Heal Force Neofuge 15R, Shanghai, China) at 12,000× *g* for 10 min to collect the supernatant.

Mycelia and media were analyzed by ultra-performance liquid chromatography-photodiode array detector (UPLC-PDA) and UPLC-tandem mass spectrometry (UPLC-MS/MS). UPLC analysis was conducted on a Waters ACQUITY UPLC system equipped with a PDA detector (Waters, Milford, MA, USA) with an ACQUITY BEH C18 column (2.1 mm × 100 mm, 1.7 μm). The flow rate was 0.3 mL/min. Mobile phases consisted of A, B and C, which were 0.1% formic acid, water, and acetonitrile, respectively. The gradient procedure was run as follows: A is always kept at 10%, 35% C for 3 min, from 35% C to 70% C in 15 min, then from 70% C to 90% C in 5 min, and lastly, kept at 35% C for 3 min. Ultraviolet absorbance was detected at 200 to 600 nm wavelength. The column temperature was 40 °C. The injection volume was 2 μL.

UPLC-MS/MS analysis was operated on a Waters ACQUITY UPLC system with Xevo tandem quadrupole mass spectrometer (Waters, Milford, MA, USA). The UPLC conditions were the same as described above. UPLC and mass spectrometry were performed by the methods established in our laboratory [28].

Emodin (Sigma-Aldrich, Shanghai, China), physcion (Sigma-Aldrich, Shanghai, China), and flavoglaucin (Biobiopha, Yunnan, China) were used as standards.

### 2.8. Comparison of Emodin and Flavoglaucin Gene Clusters

The function of genes from emodin (*A2490*) and flavoglaucin (*A7192*) gene clusters was predicted using NCBI BLASTP [20]. The alignment and visualization of homologous gene clusters in *E*. *cristatum* E1 and other known clusters were carried out using Clinker [29].

## 3. Results

### 3.1. The Basic Information of Whole Genome

**Figure 2:**
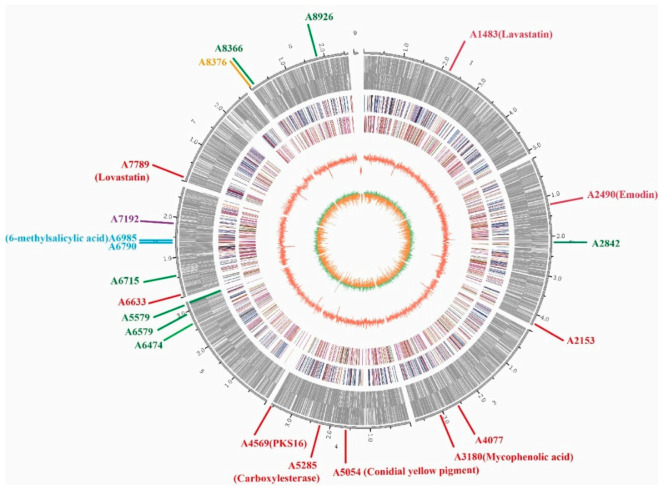
Genome map of *Eurotium cristatum* E1. From the outside to the inside: the first circle is the genome contig; the second circle is the gene density of the sense strand; the third circle is the gene density of the antisense strand; the fourth and fifth circles are the KOG annotation results; the sixth circle is the GC content calculated with 2000 bp as a window on each contig; the seventh circle is the GC offset. The polyketide synthase (PKS) and nonribosomal peptide synthase (NRPS) genes were shown in the genome map of *E*. *cristatum* E1. The red represents PKS gene. The green represents NRPS gene. The blue represents hybrid PKS and NRPS gene. The purple and orange represent indole—PKS and indole—NRPS gene, respectively.

The whole genome of *E*. *cristatum* E1 was sequenced by the combination of third-generation sequencing (TGS) and next-generation sequencing (NGS) technology. A total of 6577 Mb raw sequence data were generated. The total assembly size of the genome was 28.06 Mb, which was assembled into 9 contigs, with an N50 length of 3.56 Mb (Figure 2, Table 1). A total of 9226 genes were predicted via ab initio and homology-based analyses by the Augustus program (version 2.7). The total sequence of these coding genes was 14.29 Mb, accounting for 50.93% of the total genome sequence, with an average coding length of 1549 bp, and the GC content was 49.68% (Table 1).

The gene functional annotation was carried out using the Clusters of Eukaryotic Orthologous Groups (KOG) database. KOG database is a direct homology database for eukaryotes. It defines four main functional categories: cellular processes, information storage/processing, metabolism, and function poorly characterized, which are further subdivided into 25 categories [30]. The amino acid sequences of genes in the genome were compared with the KOG database by the Diamond software to obtain the functional annotation, and the result is shown in Supplementary Figure S1. The genes in the cellular processes and signaling were mainly annotated to O (posttranslational modification, protein turnover, chaperones, 225), U (intracellular trafficking, secretion, and vesicular transport, 125), T (signal transduction mechanisms, 123). The genes involved in information storage/processing were mainly related to translation, ribosomal structure, and biogenesis (J, 204). The metabolism analysis suggested that the genes were mainly attributed to C (energy production and conversion, 187) and E (amino acid transport and metabolism, 179). KOG analysis showed that about 844 (37.5%) of the 2248 genes assigned to KOG functional categories were involved in metabolism.

### 3.2. Phylogenetic Relationships and Comparative Genomic Analysis

A phylogenetic tree was constructed based on the concatenated multiple sequence alignments (MSA) of partial β-tubulin (*BenA*), calmodulin (*CaM*), and RNA polymerase II second largest subunit (*RPB2*) genes, and identified E1 as *Eurotium cristatum* (Supplementary Figure S2a). This agrees with the taxonomic assignment based on morphological characterization profiling (Supplementary Figure S2b). Furthermore, phylogenetic results confirm that the sequenced strain E1 is genetically closer to *Eurotium chevalieri*.

**Figure 3:**
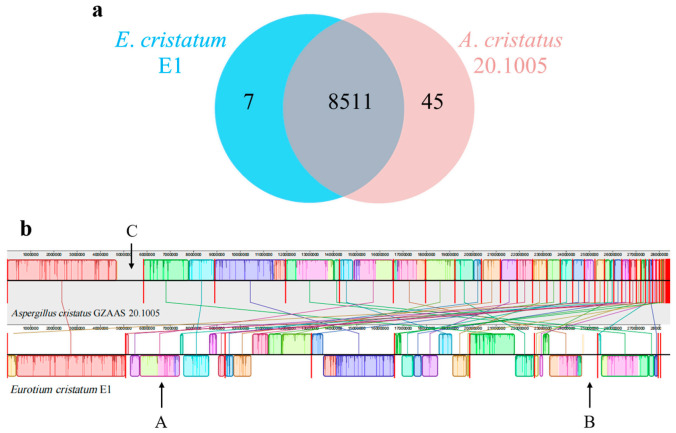
Comparative analysis of *Eurotium cristatum* E1 and *Aspergillus cristatus* GZAAS 20.1005. (**a**) Venn diagram illustrating the overlap of shared (core) and strain-specific (accessory) protein coding genes in the genomes of *E*. *cristatum* E1 and *A*. *cristatus* GZAAS 20.1005. (**b**) Each contiguously colored box is a locally collinear block (LCB), represent homologous regions with another genome without rearrangement. Lines between two genomes trace each orthologous LCB through every genome. LCBs below a genome’s center line are in the reverse complement orientation relative to the reference genome. The bold red lines represent the boundary of contig.

In 2013, the whole genome of *Aspergillus cristatus* (*A*. *cristatus* GZAAS 20.1005, GenBank: GCA_001717485.1) was reported for the first time [31]. The size of the whole genome is 28.45 Mb and is assembled into 68 scaffolds. By comparing the published genome of *A*. *cristatus* GZAAS 20.1005, it was found that the basic characteristics of the two genomes were similar; however, slight differences may also be possible among variations (Table 1). Genome-wide comparison of orthologous genes analysis of E1 and GZAAS 20.1005 is shown in Figure 3a. Two strains shared 8511 orthologous gene clusters. Notably, while the overall number of predicted genes was close for both strains (Table 1), there were 7 gene clusters unique to strain E1 and 45 clusters unique to strain 20.1005 (Figure 3a), suggestive of potentially relevant differences between the two strains. 

Then, the collinearity analysis between *E*. *cristatum* E1 and *A*. *cristatus* GZAAS 20.1005 was performed by MAUVE. Results showed that the E1 genome was highly homologous to the GZAAS 20.1005 genome, but there were still many sequence rearrangement events (Figure 3b). These events were illustrated by the crossing lines that link locally collinear blocks (LCBs), which were calculated by MAUVE to identify conserved segments that appear to be internally free from genome rearrangements. The blocks below the center line in the E1 genome suggest the presence of inversion and translocation, such as A LCBs, between two genomes. Moreover, the insertions and deletions occurred in D and E LCBs; while the insertion and inversion of short gene fragments implied that genetic recombination might have occurred during the evolution process of two strains.

### 3.3. Analysis the Biosynthesis Gene Clusters of Secondary Metabolites

Genes that participate in the same secondary metabolic pathway typically reside next to each other in fungal genomes and form a biosynthetic gene cluster (BGC). BGC contains genes encoding all enzymes that are required to produce an SM as well as pathway-specific regulatory genes. The use of the antibiotics and Secondary Metabolite Analysis Shell (antiSMASH, 5.0) enabled the prediction of 34 BGCs potentially capable of producing a diverse array of metabolites (Table 2). Only 6 of these BGCs share any homology to those deposited in the “Minimum Information about a Biosynthetic Gene Cluster” (MIBiG) database, suggesting *E*. *cristatum* may possess a unique and unknown secondary metabolite production mechanism.

The predicted SM clusters are defined according to their “backbone enzymes”, which can generate the carbon skeleton of the putative SM. In *E*. *cristatum* E1, 34 BGCs can be classified into 6 kinds of categories, non-ribosomal peptide synthetase (NRPS) clusters, type I polyketide synthase (PKS) clusters, terpene clusters, hybrid clusters, siderophore clusters and other clusters, respectively. Ten BGCs contain a gene coding for PKS enzymes, 7 BGCs contain a gene coding for NRPS enzymes and 3 BGCs are hybrid clusters containing genes coding for both PKS or NRPS enzymes. In total, twenty BGCs are related to PKS or NRPS. These results suggest that *E*. *cristatum* E1 has the potential ability to produce abundant secondary metabolites, especially polyketides and non-ribosomal peptides.

### 3.4. Genomic Distribution of PKS Genes

Previous studies on secondary metabolites produced by *E*. *cristatum* have shown that *E*. *cristatum* can produce many kinds of polyketides. PKS is the key enzyme that catalyzes the biosynthesis of polyketides. Fungal PKSs are very large multi-enzymes and contain different enzymatic domains and each domain is responsible for a different function or reaction. Nevertheless, the minimal structure of PKS only requires the KS, AT, and ACP domains [32,33]. Fungal PKS belongs to the iterative type I PKS and has a set of iteratively acting domains responsible for the catalysis of every cycle of polyketide chain elongation. 

**Figure 4:**
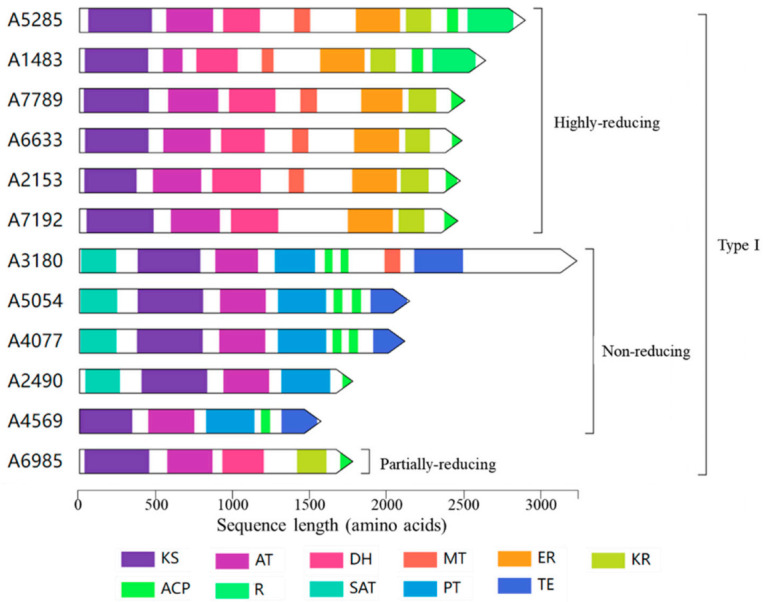
Domain arrangements of twelve PKS genes in the *Eurotium cristatum* E1 genome. According to the reduction degree, fungal PKS enzymes can be further classified into highly reducing PKS (HR-PKS, with DH, KR, and ER domains), partially reducing PKS (PR-PKS, no ER domains), and non-reducing PKS (NR-PKS, no DH, KR and ER domains). β-ketosynthase: KS; Acyltransferase: AT; Dehydratase: DH; Methyl transferase: MT; Enoylreductase: ER; Ketoacyl reductase: KR; Acyl carrier protein: ACP; Reductase: R; Starter unit ACP transacylase: SAT; Product template: PT; Thioesterase: TE.

The above analysis of *E*. *cristatum* E1 genome revealed that this fungus encodes 12 polyketide synthases (PKS, Table 3 and Figure 2), of which 6 are highly reducing (HR-PKS, synthases with enoyl-reductase, keto-reductase, and dehydratase domains), 5 non-reducing (NR-PKS, synthases with no reducing domains), and 1 partially reducing (PR-PKS, synthases with no ER domains) (Figure 4).

The homologous genes of 12 PKS genes were obtained by NCBI BlastP software and are shown in Table 3. However, only two genes, *A6985* and *A7192*, were found with highly similar homologous sequences at the amino acid level, 88% and 93%, respectively. *A6985* is responsible for the biosynthesis of 6-methylsalicylic acid, and *A7192* is involved in the biosynthesis of flavoglaucin and its congeners. Other PKSs showed limited sequence homology against current characterized PKSs. This indicated that many potential polyketides have not been discovered in *E*. *cristatum* E1. Much more attention should be paid to explore the function of uncharacterized PKSs, which is meaningful for understanding the biological characteristics of *E*. *cristatum* E1.

### 3.5. The Relative Expression Level Analysis of PKS Genes

Although 12 PKS genes in *E*. *cristatum* E1 have been identified, it is not known whether these genes can synthesize the related secondary products. Therefore, quantitative real-time PCR (qRT-PCR) was performed to measure the expression level of the predicted PKS genes. The results revealed that all genes were active, but the expression level of these genes was significantly different (Supplementary Figure S3). The expression level of the *A7789* (lovastatin) gene was the highest among all the PKS genes. Furthermore, the *A2490* (Emodin) and *A7192* (flavoglaucin) genes were also highly expressed. These results indicate that the amount of related secondary products synthesized by three genes was very high. The expression level of *A3180* was the lowest.

### 3.6. Detection of Secondary Metabolites Produced by Eurotium cristatum E1

Since all the 12 predicted PKS genes were transcribed, ultra-performance liquid chromatography (UPLC) and UPLC-tandem mass spectrometry (UPLC-MS/MS) were performed to identify the secondary metabolites produced by *E*. *cristatum* E1. Ten metabolites were found and identified, including citreorosein, emodin, physcion, echinulin, isoaspergin, dihydroauroglaucin, iso-dihydroauroglaucin, aspergin, flavoglaucin, and auroglaucin (Supplementary Figure S4). The identification of emodin, physcion, and flavoglaucin was confirmed by comparison with standards, and other compounds were identified by the comparison of their spectral data with those reported in the literature (Table 4, Supplementary Figures S5 and S6).

According to the predicted results of the function of PKS genes and their gene clusters, *E*. *cristatum* E1 can also produce many potential compounds such as lovastatin, 6-methylsalicylic acid, mycophenolic acid, but these compounds were not detected, so the secondary metabolites produced by *E*. *cristatum* need to be further studied.

### 3.7. Putative Emodin Biosynthetic Gene Cluster in Eurotium cristatum E1

Because of its very early discovery as a fungal metabolite and its structural proof by synthesis in 1924, emodin, an anthraquinone compound, plays a central role in the secondary metabolites of fungi [34]. Emodin is a polyketide and is synthesized by the regulation of NR-PKS and its cluster. In addition, a large number of studies have found that emodin is only an intermediate in this pathway, which enables the synthesis of multiple different end products, such as geodin, ravenelin, secalonic acids, monodictyphenone, etc. [35,36,37,38].

**Figure 5:**
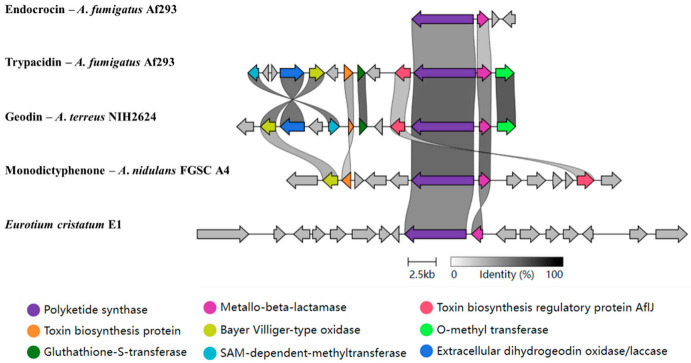
Comparison of emodin gene cluster in *Eurotium cristatum* E1 and other known species. The gene cluster used for comparison are from *A*. *fumigatus* Af293, *A*. *terreus* NIH2624, and *A*. *nidulans* FGSC A4, which have a complete emodin-related gene cluster. Each gene is indicated by an arrow. The homologous genes are shown in the same color, and other genes in gray. The genes of *A2490* gene cluster in *E*. *cristatum* E1 from left to right: *A2499*, *A2498*, *A2497*, *A2495*, *A2494*, *A2493*, *A2492*, *A2491*, *A2490*, *A2489*, *A2488*, *A2487*, *A2486*, *A2484*, *A2483*, *A2482*, *A2481*.

Only three intermediates of the emodin pathway, emodin, citreorosein, and physcion, were discovered in *E*. *cristatum* E1. In our previous study, *A2490* was proposed to regulate the biosynthesis of monodictyphenone, but at present, this compound was not found in E1. Thus, bioinformatics tools were used to speculate possible compounds, the function of genes in the *A2490* gene cluster was predicted (as shown in Table S3). The length of the *A2490* gene cluster was 44.8 kb, which contains 17 genes. Then, this cluster was compared with the known emodin-related gene clusters reported in previous reports. Only two genes, *A2489* and *A2490*, encoding metallo-beta-lactamase domain protein and polyketide synthase, found homologous genes (as shown in Figure 5). This consequence cannot confirm which end products can be synthesized by *E*. *cristatum* E1. Therefore, further research is needed on the end products produced by *E*. *cristatum* E1.

### 3.8. Putative Flavoglaucin Biosynthetic Gene Cluster in Eurotium cristatum E1

**Figure 6:**
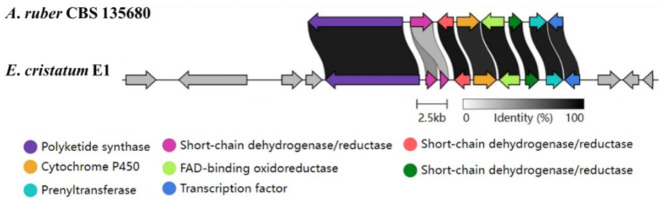
The comparison of flavoglaucin gene cluster in *Eurotium cristatum* E1 and *fog* cluster in *A*. *ruber* CBS 135680. Each gene is indicated by an arrow. The homologous genes are shown in the same color, and other genes in gray. The genes of *A7192* gene cluster in *Eurotium cristatum* E1 from left to right: *A7187*, *A7189*, *A7190*, *A7191*, *A7192*, *A7193*, *A7194*, *A7195*, *A7196*, *A7197*, *A7198*, *A7200*, *A7202*, *A7203*, *A7204*, *A7205*.

Flavoglaucin and its derivatives are a type of benzaldehyde derivatives that contain a C7 alkyl side chain with several double bonds or oxygenated substituents in their basic skeletons. Feeding experiments in 1978 confirmed that the biosynthesis of flavoglaucin is regulated by PKS and modified by isoprenylation [39]. The proposed biosynthetic pathway of flavoglaucin and its congeners was published in 2020 [40]. It has been discovered these compounds have many functions, such as antioxidant and antitumor activity [41]. The predicted function of PKS genes suggests that the *A7192* gene in *E*. *cristatum* E1 may be responsible for the biosynthesis of these compounds. Furthermore, the function of genes in the *A2490* gene cluster was predicted (as shown in Table S4). The length of the *A7192* gene cluster was 49.7 kb, which contains 16 genes. Then, the comparison between this cluster and the known flavoglaucin gene cluster was performed [40]. Pairwise amino acid alignments with the predicted proteins in this BGC revealed a well-conserved PKS, P450, prenyltransferase, FAD-binding oxidoreductase, a transcription factor as well as three short-chain dehydrogenases. The length and the number of genes involved in the two gene clusters were very different (Figure 6). However, the cultivation of E1 and UPLC analysis confirmed its capability to produce flavoglaucin and its derivatives. Therefore, further studies are necessary to confirm whether the biosynthesis pathway of flavoglaucin and its derivatives in *E*. *cristatum* E1 is consistent with the reported pathway.

## 4. Discussion

In this study, genome-wide sequencing was performed on *E*. *cristatum* E1, which is the dominant microorganism involved in a traditional fermented food—Fuzhuan brick tea. The resulting assembly was 28.06 Mb and comprised of 9 contigs. Analysis of the KOG functional classification of the proteins indicated that among the 2248 genes, 844 (37.5%) genes were assigned to functional annotations with significant biological significance. The results also showed that the genes were outstanding in energy production and conversion, amino acid transport and metabolism, carbohydrate transport and metabolism, secondary metabolites biosynthesis, transport and catabolism, etc., which might suggest the advantages of this strain for producing abundant metabolites.

The cost of genome sequencing has decreased rapidly, due to the invention of next-generation sequencing (NGS) technologies, e.g., the Pacific Biosciences Single Molecule Real-Time (PacBio SMRT) platform [42]. Particularly, the Illumina short-read-based genome assemblies frequently missed large fragments of giant genes encoding PKS and NRPS, which play an important key role in studying the secondary metabolites of fungi [43]. However, next-generation sequencing technologies have solved this problem and allowed researchers to gain a deeper understanding of the molecular and genetic mechanisms of fungi [44]. To date, there is only one complete annotated genome of *E*. *cristatum* available in the NCBI database. The whole genome of *A*. *cristatus* (*A*. *cristatus* GZAAS 20.1005, GenBank: GCA_001717485.1), published in 2013, was obtained by the Illumina Hiseq™ 2000 system and assembled into 68 scaffolds [31]. The number of scaffolds is much higher than that of E1, which fits the longer reading advantage of NGS [36]. In addition, the comparative analysis of collinearity between genomes not only revealed the rearrangement, inversion, and translocation between two genomes but also found the absence of long fragments in both genomes. These results implied that genetic recombination may have occurred during the evolution process of the two strains.

*E*. *cristatum* is a kind of filamentous fungi, which is renowned for producing a wide variety of secondary metabolites with various bioactivities [11]. With the rapid development of bioinformatics, genome mining for screening gene clusters is a new method for investigating the SMs produced by a fungus [45]. BGC analysis of *E*. *cristatum* E1 discovered, out of 34 BGCs, that only 6 BGCs showed different levels of similarity with known gene clusters. Furthermore, only 3 BGCs showed more than 80% similarity to known BGCs. Thus, *E*. *cristatum* E1 might present a higher potential for producing new metabolites. Among the 34 BGCs, 20 BGCs were related to PKS or NRPS, which might reflect the potential of this fungi to produce various polyketides and non-ribosomal peptides.

Polyketides are very important secondary metabolites with great diversity in their structures, functional diversities, and a wide range of biological activities, which are regulated by PKS [10]. It is worth mentioning that the number of PKS genes in *E*. *cristatum* E1 was lower than that in other *Aspergillus* species (Supplementary Figure S7) [46,47]. It is very strange that there are only 12 PKS genes in *E*. *cristatum* E1 because based on previous studies, *E*. *cristatum* E1 has a stronger ability to produce polyketides such as emodin and flavoglaucin [12,14]. In addition, the qRT-PCR results further demonstrated that these genes were expressed and had the ability to synthesize related compounds.

However, the above results only demonstrated that *E*. *cristatum* E1 can produce these compounds at the genomic level. Subsequently, the secondary metabolites produced by *E*. *cristatum* E1 were analyzed by UPLC and UPLC-MS/MS. Ten compounds were identified, but only two compounds, emodin and flavoglaucin, have been found in the PKS genes that might be responsible for their synthesis. Furthermore, based on the bioinformatics analysis results, compounds such as lovastatin, 6-methylsalicylic acid, mycophenolic acid, etc., were predicted. However, compared to the standards, lovastatin and mycophenolic acid could not be detected in the above UPLC and UPLC-MS/MS analysis; therefore, further research is needed to verify the secondary metabolites produced by *E*. *cristatum* E1.

Emodin, as an important metabolite, has been studied since 1924. It has many functions, such as antibacterial, antiviral, antioxidant, etc. [34]. Emodin is only an intermediate, it can be converted into different end-products, depending on the *Aspergillus* sp. [48]. However, only emodin, citreorosein, and physcion were isolated from *E*. *cristatum* E1. In the emodin biosynthesis pathway, citreorosein and physcion are intermediate products, which are produced after the synthesis of emodin. Different compounds are then synthesized through a series of other reactions [49]. Compared with known emodin-related gene clusters, only *A2489* and *A2490* had found their homologous genes; while, according to previous studies, the presence of these two genes can only produce emodin [36,37]. From the gene clusters map, the length of the emodin gene cluster is much longer than other clusters. It contains 17 genes, whereas other clusters contain at most 13 genes, and only four genes are involved in the endocrocin gene cluster. The similarity between the *A2490* gene cluster and the known gene cluster is very low. Whether citreorosein and physcion are intermediate products or end-products produced by *E*. *cristatum* E1 needs further study. In general, combined with the results of gene cluster alignment and secondary metabolites analysis, there may be a different emodin-related pathway that exists in *E*. *cristatum* E1. Further studies are needed to confirm this hypothesis.

Flavoglaucin is a class of benzaldehyde derivatives that contain a variety of derivatives, which occur widely in *Aspergillus* sp. [39]. It was discovered in the last century, but the biosynthetic pathway was only reported in 2020 [50]. In 2020, the biosynthetic gene cluster of flavoglaucin in *Aspergillus ruber* was proposed [40,50]. Furthermore, the comparison between the proposed BGC in E1 and the known flavoglaucin gene cluster showed that the number of genes varies enormously (16 vs. 8). However, the similarity of PKS genes and the eight genes downstream of BGC at the amino acid level was more than 85%. In addition, *A*. *ruber* can produce six kinds of flavoglaucin and congeners, including isoaspergin, dihydroauroglaucin, aspergin, flavoglaucin, auroglaucin, and 2-(1′,5′-heptadienyl)-3,6-dihydroxy-5-(3″-methyl-2″-butenyl) benzaldehyde [40]. However, the analysis of secondary metabolites of *E*. *cristatum* E1 found that it cannot produce the last compound, but can produce iso-dihydroauroglaucin. Whether these differences will lead to different biosynthetic pathways in *E*. *cristatum* E1 requires further study. The putative emodin and flavoglaucin gene clusters were proposed, but large differences were found by comparison with known gene clusters. However, some related compounds were identified from *E*. *cristatum* E1. Furthermore, the biosynthetic pathways of emodin and flavoglaucin need to be proved by several approaches, such as gene knockout, in vitro reaction, and heterologous expression.

Although these results indicate that compounds detected in *E*. *cristatum* corresponded to the gene function analysis, the identification of other compounds and PKS genes still needs to be further studied. Firstly, further isolation and identification of secondary metabolites in *E*. *cristatum* E1 are needed. Secondly, the development of new bioinformatic tools will help in the identification and annotation of new gene clusters. Moreover, using molecular biological methods, such as gene knockout, is a direct approach to determine the function of genes. Overall, these previous results might be significantly important for understanding *E*. *cristatum* E1. 

In this work, a combined approach beginning with genome mining for screening, followed by transcript-level expression analysis, and the identification of products from wild-type fungi, was used to systematically study the polyketides produced by *E*. *cristatum* E1. The function of PKS genes was analyzed after obtaining genomic information through whole-genome sequencing, and the possible compounds were speculated. Ten compounds were identified from this strain, in which the biosynthetic gene and its gene cluster responsible for two compounds (emodin and flavoglaucin) were identified. These results demonstrate a higher potential in discovering novel secondary metabolites from *E*. *cristatum* E1, and also provide some guidelines into the use of combining bioinformatics tools and the approach of isolating and identifying compounds in the discovery of secondary metabolites. This will provide a good basis for systematically mining SMs resources of *Eurotium cristatum* and broadening its application fields.

## Figures and Tables

**Table 1:** General feature of *E*. *cristatum* E1 genome and *A*. *cristatus* GZAAS 20.1005 genome.

Genome	*E*. *cristatum* E1	*A*. *cristatus* GZAAS 20.1005
General information		
Size (Mb)	28.06	27.9
Number of contigs/scaffolds	9	68
N50 (bp)	3,564,179	2,308,221
G + C content (%)	49.72	49.86
Coding (%)	50.93	51.92
Protein-coding genes	9226	10,136
Mean gene length (bp)	1549	1573

**Table 2:** Secondary metabolites gene clusters in *Eurotium cristatum* E1.

Cluster	Contig	Gene Cluster Type	Backbone Enzymes	Predicted Products	Chemical Formulation	Similarity
BGC1	Contig1	t1pks	PKS	-		
BGC2	Contig2	t1pks	PKS	TAN-1612	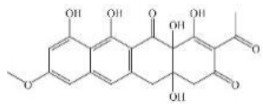	60%
BGC3	Contig2	t1pks	PKS	-		
BGC4	Contig3	t1pks	PKS	-		
BGC5	Contig3	t1pks	PKS	-		
BGC6	Contig4	t1pks	PKS	Naphthopyrone	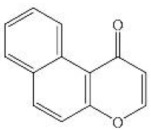	100%
BGC7	Contig4	t1pks	PKS	-		
BGC8	Contig4	t1pks	PKS	-		
BGC9	Contig6	t1pks	PKS	-		
BGC10	Contig7	t1pks	PKS	Cornexistin	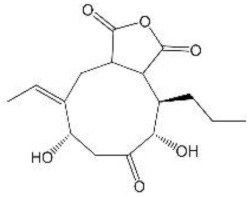	9%
BGC11	Contig2	nrps	NRPS	-		
BGC12	Contig5	nrps	NRPS	-		
BGC13	Contig5	nrps	NRPS	-		
BGC14	Contig5	nrps	NRPS	-		
BGC15	Contig6	nrps	NRPS	-		
BGC16	Contig8	nrps	NRPS	-		
BGC17	Contig8	nrps	NRPS	-		
BGC18	Contig6	t1pks-nrps	PKS- NRPS	6-methylsalicylic acid	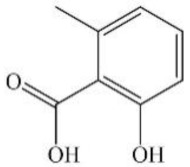	100%
BGC19	Contig6	Indole-t1pks	PKS	Neurosporin A		20%
BGC20	Contig8	Indole-nrps	NRPS	-		
BGC21	Contig2	Terpene		-		
BGC22	Contig5	Terpene		-		
BGC23	Contig5	Terpene		-		
BGC24	Contig7	Terpene		-		
BGC25	Contig8	Terpene		Clavaric acid	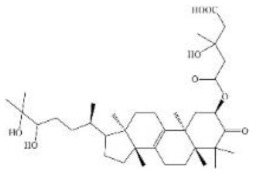	66%
BGC26	Contig3	Siderophore		-		
BGC27	Contig3	Other		-		
BGC28	Contig3	Other		-		
BGC29	Contig3	Other		-		
BGC30	Contig3	Other		-		
BGC31	Contig4	Other		-		
BGC32	Contig5	Other		-		
BGC33	Contig6	Other		-		
BGC34	Contig8	Other		-		

“-” Represents unknown products.

**Table 3:** Summary of putative PKS genes in the *Eurotium cristatum* E1 genome.

Type	Number	PKS ID	Amino Acids	Homologs and Related Description	Sequence Identity	BGC ID—Scaffold
hrPKS						
	1	A1483	2643	Lovastatin diketide synthase LovF (GenBank: GAQ02995.1)	54%	BGC01—scaffold1
	2	A2153	2478	Beta-ketoacyl synthase (GenBank: CDM30483.1)	56%	BGC03—scaffold2
	3	A5285	2901	Carboxylesterase type B (GenBank: OOO04444.1)	56%	BGC08—scaffold4
	4	A6633	2489	Acyl transferase/acyl hydrolase/lysophospholipase (GenBank: KGO38831.1)	56%	BGC09—scaffold6
	5	A7192	2462	polyketide synthase (GenBank: EYE95336.1)	93%	BGC19—scaffold6
	6	A7789	2508	Lovastatin diketide synthase LovF (GenBank: GAQ05228.1)	53%	BGC10—scaffold7
nrPKS						
	7	A2490	1778	Monodictyphenone synthesis protein mdpG (GenBank: Q5BH30.1)	53%	BGC02—scaffold2
	8	A3180	3237	Mycophenolic acid synthesis protein mapC (GenBank: F1DBA9.1)	46%	BGC04—scaffold3
	9	A4077	2117	Beta-ketoacyl synthase N-terminal domain family protein (GenBank: OWW34975.1)	61%	BGC05—scaffold3
	10	A4569	1571	PKS16 protein (GenBank: EYE96821.1)	76%	BGC06—scaffold4
	11	A5054	2148	Conidial yellow pigment biosynthesis polyketide synthase (GenBank: OOQ85606.1)	71%	BGC07—scaffold4
prPKS						
	12	A6985	1778	6-methylsalicylic acid synthase MsaS (GenBank: EYE91246.1)	88%	BGC18—scaffold6

**Table 4:** Molecular weights and UV–Vis spectrum of secondary metabolites produced by *Eurotium cristatum* E1.

Num	Compounds	UPLC-MS/MS			UV Chromatogram
		m/z ([M + H]^+^)	m/z ([M − H]^−^)	m/z	
1	Isoaspergin	303.141	301.164	302.152	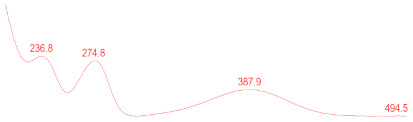
2	Dihydroauroglaucin	301.164	299.314	300.239	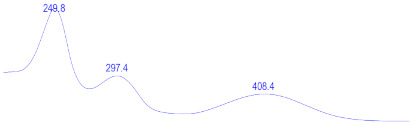
3	Iso-dihydroauroglaucin	300.59	298.932	299.761	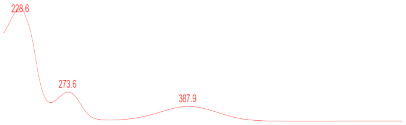
4	Aspergin	303.97	301.291	302.630	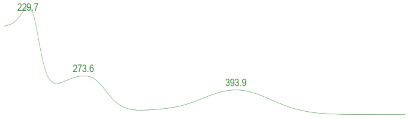
5	Flavoglaucin	304.799	302.949	303.874	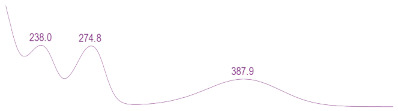
6	Auroglaucin	299.123	297.083	298.103	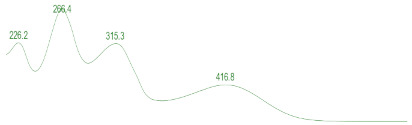
7	Citreorosein	286.688	284.902	285.795	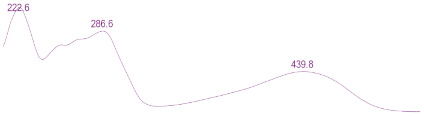
8	Emodin	270.681	268.959	269.82	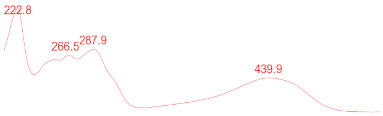
9	Physcion	285.872	283.831	284.851	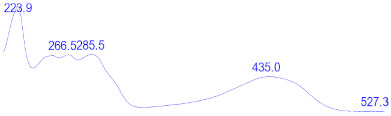
10	Echinulin	462.297	460.129	461.213	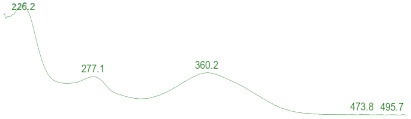

## Data Availability

External data sources used in this study are cited in article. The extracted data is available in Supplementary Material.

## Supplementary Materials

The following are available online at https://www.mdpi.com/article/10.3390/jof8020193/s1, Figure S1: KOG distribution of predicted proteins from *Eurotium cristatum* E1 genome, Figure S2: Confirmation of the taxonomic classification of *Eurotium cristatum* E1, Figure S3: Real-time RT-PCR analysis of the PKS genes in *Eurotium cristatum* E1, Figure S4: UPLC analysis of *Eurotium cristatum* E1 extracts, Figure S5: UPLC-MS analysis of *Eurotium cristatum* E1 extracts, Figure S6: ESIMS spectra in negative mode of flavoglaucin and its derivatives (1–6), citreorosein (7), emodin (8), physion (9) and echinulin (10), Figure S7: The number of PKS genes in some sequenced *Aspergillus* species, Table S1: Strains and accession information of genes used for phylogenetic tree construction, Table S2: Primers used for qRT-PCR, Table S3: Analysis of *A2490* gene cluster in *Eurotium cristatum* E1, Table S4: Analysis of *A7192* gene cluster in *Eurotium cristatum* E1.
