# Supplementary material for: Genome Mining and Analysis of PKS Genes in Eurotium cristatum E1 Isolated from Fuzhuan Brick Tea
Source: J Fungi (Basel). 2022 Feb 16;8(2):193. doi: 10.3390/jof8020193 (PMC8874483; doi:10.3390/jof8020193)
Supplement: Supplementary file 1 [file jof-08-00193-s001.zip › jof-1572868-SI.pdf]

Journal of
Fungi

MDPI

Supplementary Materials:

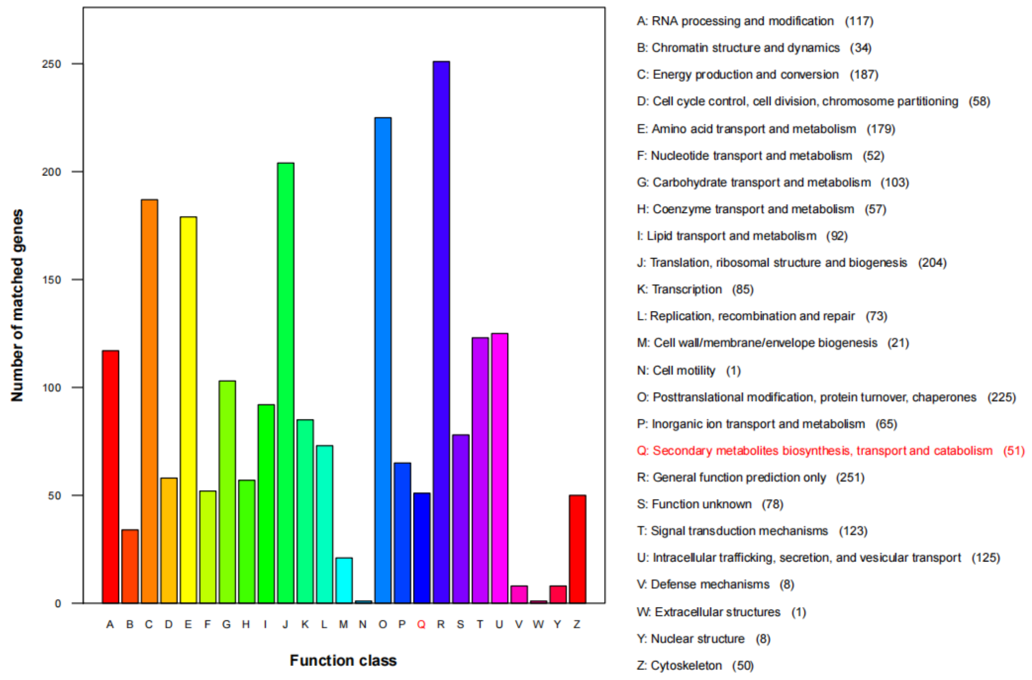

Figure S1. KOG distribution of predicted proteins from *Eurotium cristatum* E1 genome. KOG categories are as follows. M, O, T, U, V, W, Y and Z belong to cellular processes and signaling. A, B, J, K and L belong to information storage and processing. C, D, E, F, G, H, I, P and Q belong to metabolism. R and S belong to poorly characterized.

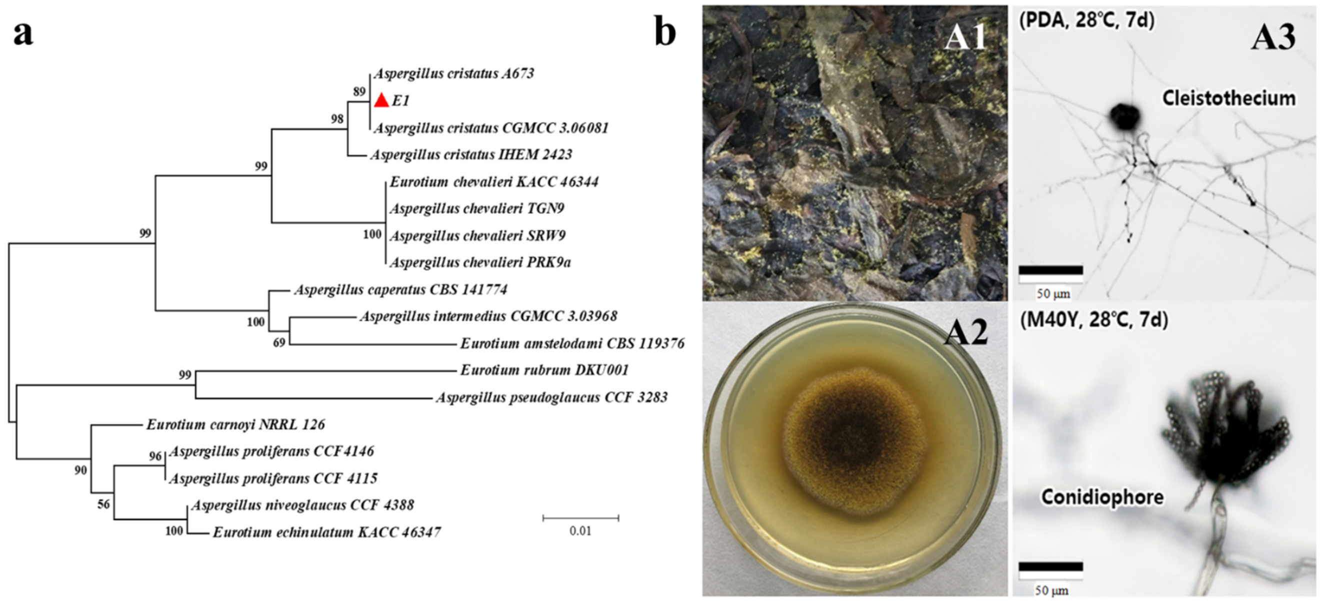

Figure S2. Confirmation of the taxonomic classification of *Eurotium cristatum* E1. (**a**) Phylogenetic tree. *E. cristatum* E1 sequence was compared with fragments of the partial β-tubulin (*BenA*), calmodulin (*CaM*) and RNA polymerase II second largest subunit (*RPB2*) genes of 18 *Aspergillus* species and *Eurotium* species retrieved from GenBank. The phylogenetic tree was constructed by MEGA 5.05 based on Neighbor-Joining method. Detail information of the sequences used in phylogenetic tree can be found in Supplementary Table S1. (**b**) Morphological characteristics of *E. cristatum* E1 isolated from Fuzhuan brick tea. (A1) A piece of Fuzhuan brick-tea with "Jinhua" (yellow dots) visible to the naked eye; (A2) Single colony grown on PDA agar in a 7 cm petri dish at 28 °C for 9 days; (A3) Microscopic image of cleistothecium and conidiophore (×200).

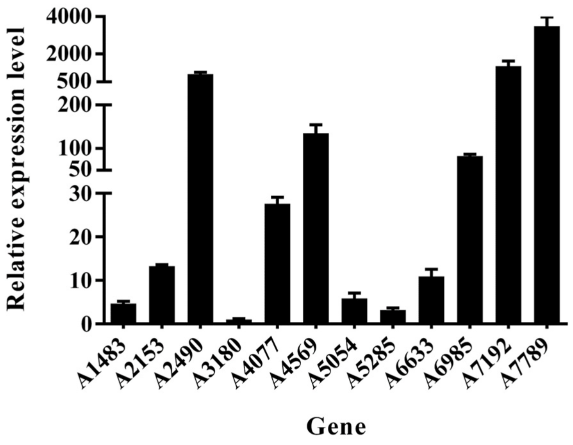

Figure S3. Real-time RT-PCR analysis of the PKS genes in *Eurotium cristatum* E1. Relative expression of the PKS genes in *E. cristatum* E1 was incubated in PDA medium for 5 days at 28 °C. *β-actin* was used as the reference gene. The error bars indicate the standard deviations of three independent repetitions.

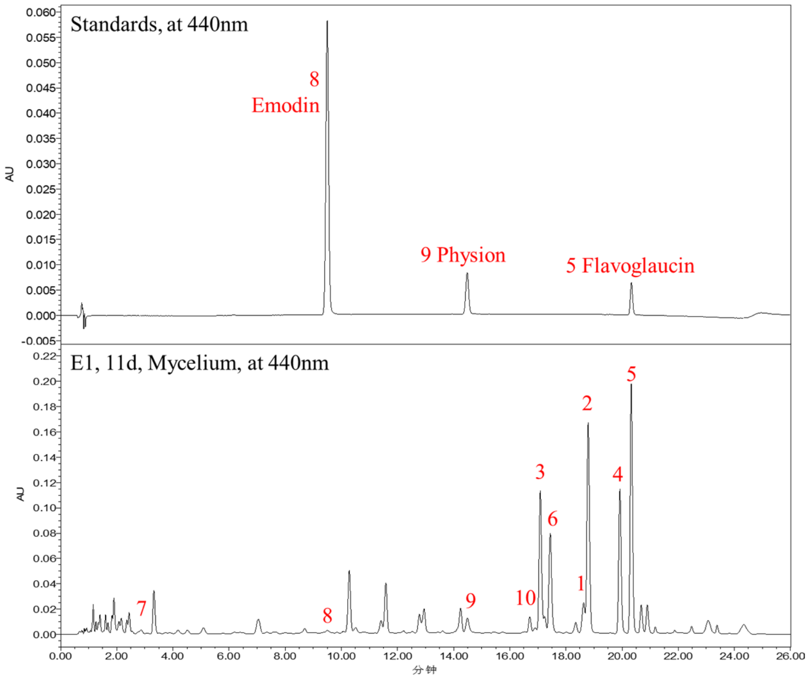

Figure S4. UPLC analysis of *Eurotium cristatum* E1 extracts. The strains were cultivated in PDA medium for 11 days at 28 °C.

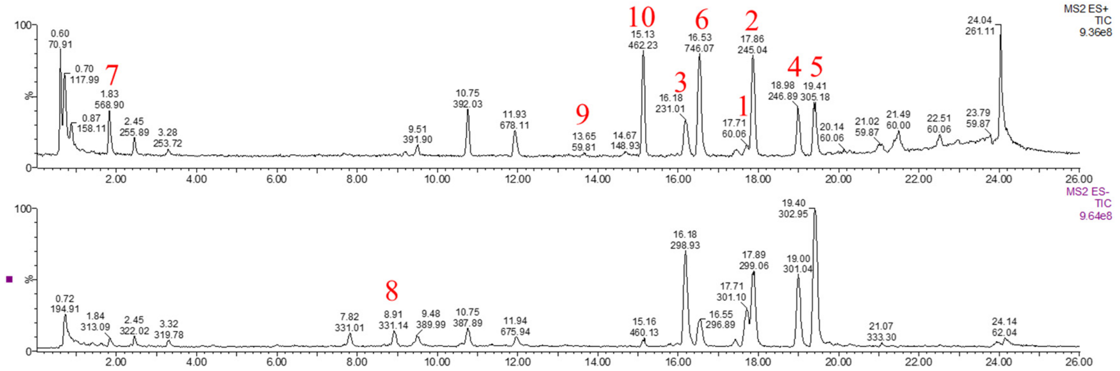

Figure S5. UPLC-MS analysis of *Eurotium cristatum* E1 extracts. The strains were cultivated in PDA medium for 11 days at 28 °C.

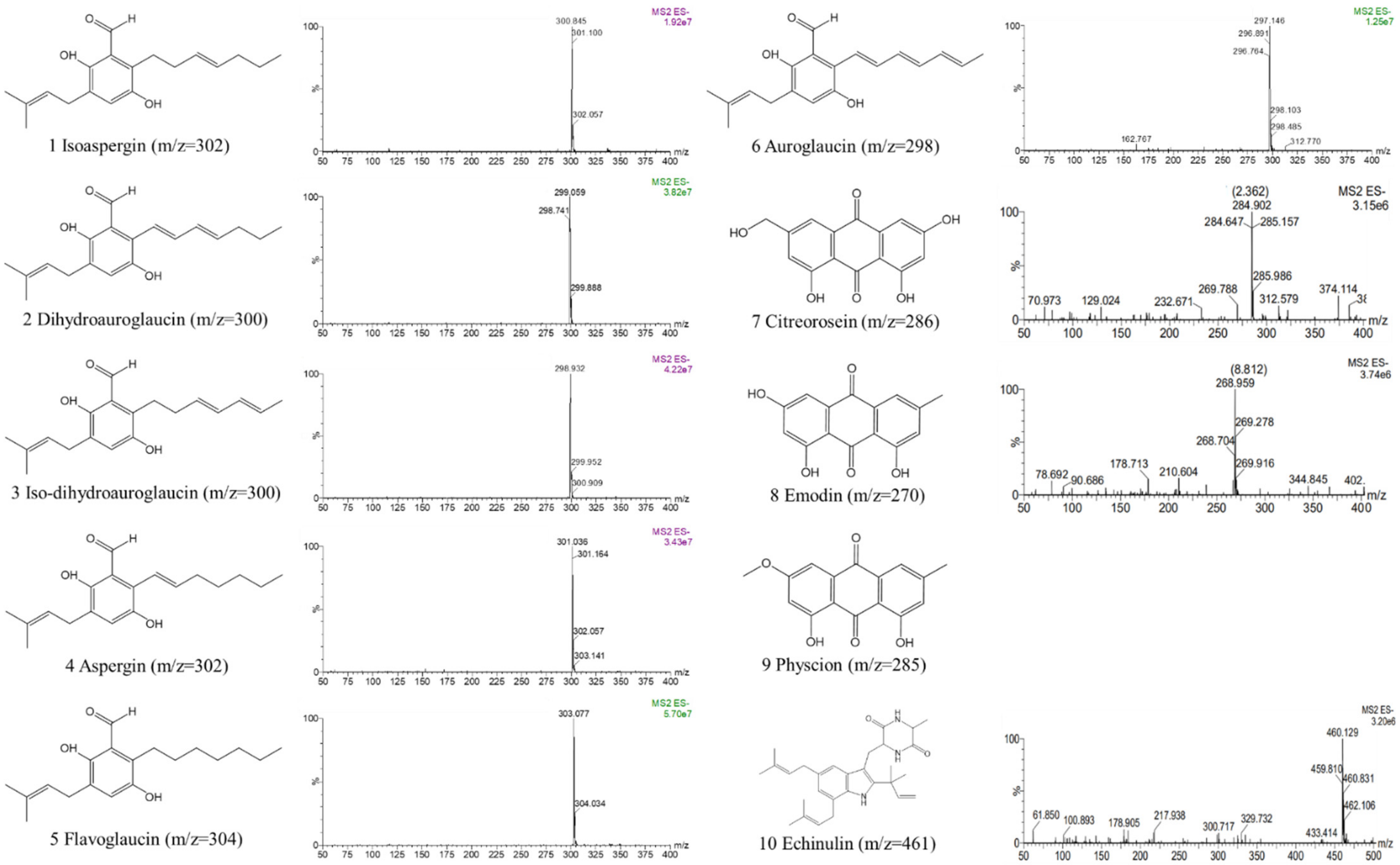

Figure S6. ESIMS spectra in negative mode of flavoglaucin and its derivatives (1-6), citreorosein (7), emodin (8), physion (9) and echinulin (10).

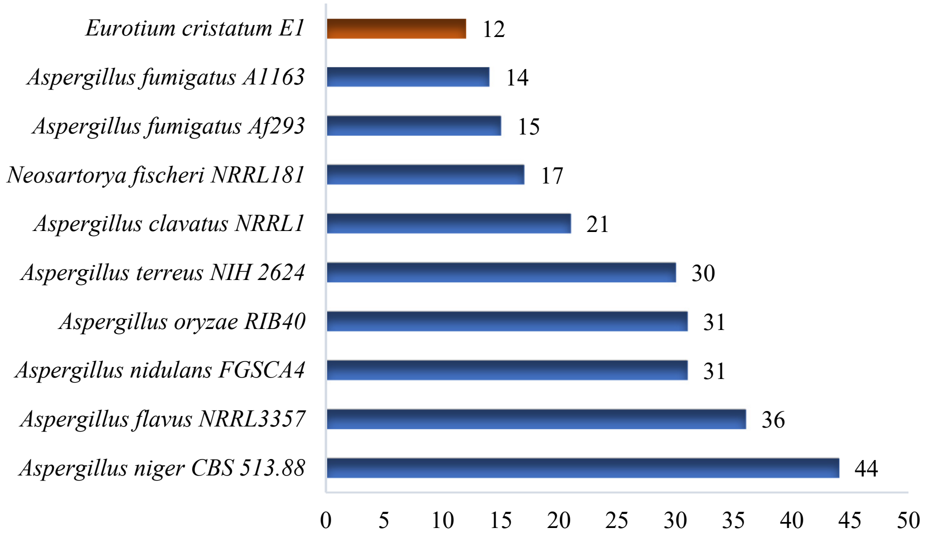

Figure S7. The number of PKS genes in some sequenced *Aspergillus* species.

Table S1. Strains and accession information of genes used for phylogenetic tree construction.

Strain	Accession		
	β-tubulin	*CaM*	*RPB2*
Aspergillus caperatus CBS 141774	LT671008.1	LT671009.1	LT671010.1
Aspergillus chevalieri NRRL 78	EF651911.1	EF652002.1	EF651954.1
Aspergillus chevalieri NRRL 4755	EF651913.1	EF652004.1	EF651956.1
Aspergillus cristatus CGMCC 3.06081	LT671061.1	LT671062.1	LT671063.1
Aspergillus cristatus IHEM 2423	LT671058.1	LT671059.1	LT671060.1
Eurotium cristatum NRRL 4222	EF651914.1	EF652001.1	EF651957.1
Eurotium echinulatum NRRL 131	EF651907.1	EF651998.1	EF651939.1
Eurotium herbariorum NRRL 116	EF651887.1	EF651989.1	EF651934.1
Eurotium repens NRRL 13	EF651915.1	EF652005.1	EF651950.1
Eurotium rubrum NRRL 52	EF651920.1	EF652009.1	EF651947.1
Eurotium tonophilum NRRL 5124	EF651919.1	EF652000.1	EF651969.1
Aspergillus intermedius CGMCC 3.03968	LT671082.1	LT671083.1	LT671084.1
Aspergillus proliferans CCF 4146	HE578076.1	HE650909.1	HE801304.1
Aspergillus proliferans CCF 4115	FR851855.1	HE578090.1	HE578107.1
Aspergillus pseudoglaucus CCF 3283	FR775360.2	HE974439.1	HE578110.2
Eurotium amstelodami NRRL 90	EF651897.1	EF652017.1	EF651963.1
Eurotium amstelodami NRRL 4716	EF651899.1	EF652018.1	EF651965.1
Eurotium carnoyi NRRL 126	EF651903.1	EF651985.1	EF651942.1

Table S2. Primers used for qRT-PCR.

Primers	Sequences (5′→3′)	Functions
A1483-qPCR-f	AGGTGGATGGTTGAGAAGGG	For real time RT-PCR analysis of *A1483*
A1483-qPCR-r	TTCCTCCTACCGGCTTTCTG	
A2153-qPCR-f	GTCGTGGAGAAGGAGTTGGA	For real time RT-PCR analysis of *A2153*
A2153-qPCR-r	CGAGCGGATGAGAGATTCCT	
A2490-qPCR-f	TCATTGATGACCCGGCTCTT	For real time RT-PCR analysis of *A2490*
A2490-qPCR-r	TTGCCTGGCCATAGAAGACA	
A3180-qPCR-f	CCTAGCAGTTGTGTCCCAGA	For real time RT-PCR analysis of *A3180*
A3180-qPCR-r	TCAAAATCAACAGCTGCCCC	
A4077-qPCR-f	ATGAGGGCGTTAGGTTCCAA	For real time RT-PCR analysis of *A4077*
A4077-qPCR-r	ATGAGGACCCATTTCTGCCA	
A4569-qPCR-f	TGGAGGCCAAGCGATATCAT	For real time RT-PCR analysis of *A4569*
A4569-qPCR-r	TAGACCGACAATACTCCCGC	
A5054-qPCR-f	ACCGTGAGATCAACAGTGGT	For real time RT-PCR analysis of *A5054*
A5054-qPCR-r	GTGAATAGCGGCAAGACTGG	
A5285-qPCR-f	GGAAGAAACATTGCGTGGGT	For real time RT-PCR analysis of *A5285*
A5285-qPCR-r	AAGCCTGGAAAGTCGATGGA	
A6633-qPCR-f	CCAGTGGTGAAATTGCTGCT	For real time RT-PCR analysis of *A6633*
A6633-qPCR-r	GATTGTCGTGCTGGATGGAC	
A6985-qPCR-f	GGAAGGCCAATTGACGATCC	For real time RT-PCR analysis of *A6985*
A6985-qPCR-r	CTTCGTGCCAGTTAATCCGG	
A7192-qPCR-f	CTTGGGTTCGCAGCTATCAC	For real time RT-PCR analysis of *A7192*
A7192-qPCR-r	GTTGAGCACTACGTCGACAC	
A7789-qPCR-f	TACGATCCTCATCAACGGGG	For real time RT-PCR analysis of *A7789*
A7789-qPCR-r	GAAATCATCCACGTCGCGAA	
β-actin-qPCR-f	TCTGGCACCACACATTCTACA	For real time RT-PCR analysis of *β-actin*
β-actin-qPCR-r	CGAAGACGATCTGGGTCATCT	

Table S3. Analysis of *A2490* gene cluster in *Eurotium cristatum* E1.

ORF	Size (bp/aa)	Homologs and related description	Sequence identity
A2499	4608/1535	hypothetical protein (GenBank: XP_040633901.1)	75%
A2498	1028/266	**TATA-box-binding protein** (GenBank: XP_043134035.1)	99%
A2497	1452/436	**fructosyl amino acid oxidasesarcosine oxidase** (GenBank: XP_040641941.1)	95%
A2495	1110/351	**ubiquinone/menaquinone biosynthesis-related protein** (GenBank: XP_040641942.1)	87%
A2494	1397/402	hypothetical protein (GenBank: XP_022402423.1)	92%
A2493	2139/694	**fungal-specific transcription factor** (GenBank: KAE8149832.1)	46%
A2492	993/295	**NADH-cytochrome b5 reductase-like protein** (GenBank: XP_040641945.1）	88%
A2491	669/222	**Methyltransferase** (GenBank: XP_040641946.1)	91%
A2490	5496/1778	**polyketide synthase** (GenBank: Q5BH30.1)	53%
A2489	982/308	**metallo-beta-lactamase** (GenBank: XP_040641948.1)	91%
A2488	1777/513	**pisatin demethylase** (GenBank: XP_040641950.1)	92%
A2487	1749/484	**cytochrome P450** (GenBank: XP_040641951.1)	91%
A2486	1202/359	**NAD(P)-binding protein** (GenBank: XP_040641952.1)	91%
A2484	1349/409	hypothetical protein (GenBank: XP_043134047.1)	94%
A2483	956/207	hypothetical protein (GenBank: RJE24327.1)	64%
A2482	1555/494	hypothetical protein (GenBank: XP_043134049.1)	97%
A2481	2804/878	**C6 transcription factor** (GenBank: XP_025574120.1)	60%

Table S4. Analysis of *A7192* gene cluster in *Eurotium cristatum* E1.

ORF	Size (bp/aa)	Homologs and related description	Sequence identity
A7187	1689/562	hypothetical protein (GenBank: XP_002375531.1)	48.21%
A7189	4983/1660	**Mitogen-Activated Protein (MAP) kinase** (GenBank: EYE95333.1)	95.54%
A7190	1437/478	**ammonium transporter** (GenBank: EYE95334.1)	97.06%
A7191	1158/385	**nuclear mRNA splicing factor-associated protein** (GenBank: XP_025499988.1)	81.41%
A7192	7389/2462	**polyketide synthase** (GenBank: EYE95336.1)	92.26%
A7193	822/273	**short chain dehydrogenase** (GenBank: EYE95337.1)	91.54%
A7194	612/203	**short chain dehydrogenase** (GenBank: EYE95337.1)	94.03%
A7195	861/286	**short chain dehydrogenase** (GenBank: EYE95338.1)	93.01%
A7196	1497/498	**cytochrome P450** (GenBank: EYE95339.1)	86.80%
A7197	1497/498	**FAD-binding oxidoreductase** (GenBank: EYE95340.1)	91.77%
A7198	1047/348	**short chain dehydrogenase** (GenBank: EYE95341.1)	91.95%
A7200	1308/435	**DMATS superfamily prenyltransferase** (GenBank: ATP76208.1)	90.57%
A7201	1245/414	**Transcription factor** (GenBank: EYE95343.1)	86.06%
A7202	1389/462	**tubulin beta chain** (GenBank: EYE95344.1)	99.32%
A7203	1233/410	hypothetical protein (GenBank: XP_001269171.1)	70.75%
A7204	750/249	hypothetical protein (GenBank: XP_022400343.1)	74.77%
A7205	2619/872	**protein kinase domain family protein** (GenBank: TPR03492.1)	32.93%
